# The Impact of Comorbidity on Survival in Patients With Head and Neck Squamous Cell Carcinoma: A Nationwide Case-Control Study Spanning 35 Years

**DOI:** 10.3389/fonc.2020.617184

**Published:** 2021-02-17

**Authors:** Eva Kristine Ruud Kjær, Jakob Schmidt Jensen, Kathrine Kronberg Jakobsen, Giedrius Lelkaitis, Irene Wessel, Christian von Buchwald, Christian Grønhøj

**Affiliations:** ^1^ Department of Otorhinolaryngology, Head and Neck Surgery and Audiology, University of Copenhagen, Rigshospitalet, Copenhagen, Denmark; ^2^ Department of Pathology, University of Copenhagen, Rigshospitalet, Copenhagen, Denmark; ^3^ Department of Otorhinolaryngology and Maxillofacial Surgery, Zealand University Hospital, Køge, Denmark

**Keywords:** head and neck cancer, comorbid, epidemiology, survival, Charlson Age Comorbidity Index

## Abstract

**Background:**

Comorbidity is presumed to impact survival of head and neck squamous cell cancer (HNSCC) patients. However, the prevalence and prognostic impact of comorbidity in these patients is not yet well established. The aim of this study is to outline the comorbidity burden of HNSCC patients and investigate the relation to overall survival and cancer-specific mortality.

**Methods:**

The comorbidity burden of patients registered with HNSCC in the Danish Cancer Registry between 1980 and 2014 was evaluated based on the Charlson Comorbidity Index (CCI). Patients’ risks of comorbid conditions compared to age- and gender-matched controls were estimated by odds ratios (OR). The impact of comorbidity on overall survival and cancer-specific mortality was evaluated by Cox regression and Kaplan-Meier survival analysis.

**Results:**

A total of 25,388 HNSCC patients were included (72.5% male; mean age 63.2 years at diagnosis; median follow-up 3.0 years). CCI at diagnosis was significantly higher in patients compared to controls (*p < 0.001*). The most common comorbid conditions among the patients were additional non-metastatic malignancy (10.9%) and cerebrovascular disease (7.7%). Compared to controls, patients had higher odds of metastatic malignancy (OR: 4.65; 95% CI: 4.21–5.15; *p < 0.001*), mild liver disease (OR: 6.95; 95% CI: 6.42–7.53; *p < 0.001*), and moderate-severe liver disease (OR: 7.28; 95% CI: 6.14–8.65; *p < 0.001*). The multivariate Cox analysis revealed increasing hazard ratios with increasing CCI and in coherence the Kaplan-Meier curves showed poorer overall survival and increased cancer-specific mortality in patients with higher CCI.

**Conclusion:**

HNSCC patients’ comorbidity burden was significantly greater compared to the general population and increased comorbidity was correlated with increased cancer-related mortality.

## Introduction

More than 90% of cancers in the head and neck region are squamous cell carcinomas (HNSCC) (1), a heterogeneous group of malignancies arising from the squamous mucosal lining of the oral cavity, nasopharynx, oropharynx, hypopharynx, sinonasal cavities, and larynx. Globally, the incidence of registered malignancies of the lip, oral cavity, pharynx, and larynx increased from 686,000 new cases in 2012 to 835 000 new cases in 2018 (2, 3). HNSCC is responsible for considerable mortality with an estimated 431,000 deaths in 2018 and exhibit a multifactorial etiology, largely attributed to smoking and excessive alcohol consumption, and in recent years infection with human papillomavirus (HPV) has become a well-accepted risk factor of HNSCC originating from the oropharynx (3, 4).

Medical comorbidities in HNSCC affect mortality, treatment outcome, and ability to complete therapy and adhere to follow-up regimens (5–8). The Charlson Comorbidity Index (CCI) is a method to determine prognostic comorbidity of patients in longitudinal studies and takes into consideration 17 comorbid conditions each assigned a certain weight (9, 10). The CCI approach is based on the International Classification of Diseases (ICD) and has proved to be precise in register-based studies (11, 12). Long-term data on HNSCC patients’ comorbidities and CCI development is lacking and could possibly ameliorate understanding of the medical challenges that patients face and modify follow-up strategies.

This study reports the comorbidity burden and the related survival of Danish HNSCC patients in a population-based, nation-wide setting and investigates the increase of CCI over time after HNSCC diagnosis. In addition, it reports the most prevalent comorbid conditions in patients and controls.

## Patients and Methods

This study was based on data from the Danish Cancer Registry (DCR), the central population registry (CPR), the Register of Causes of Death (RCG), and the Danish National Patient Register (NPR). DCR contains information on cancer diagnoses such as date of diagnosis, tumor location, and tumor morphology, and registration became mandatory in 1987 (13). Diagnostic classifications are coded according to the International Classification of Diseases of Oncology (ICD-O) with the use of ICD-O-3 topography and morphology codes. Cancers diagnosed in DCR have been converted from ICD-O into ICD-10. CPR contains information on vital status, gender, and date of birth linked to unique civil registration numbers, which is assigned to all Danish residents at birth or upon immigration (14). RCG contains information on cause-specific mortality of all deceased Danish residents (15). NPR comprises all registered hospital contacts in Denmark since it was established in 1977, however contains a small fraction of registrations before 1977 dating back to 1946. Each hospital contact in the NPR has a unique record number to which civil registration number, dates, medical and surgical procedures, and diagnosis codes are linked. Diagnostic information in NPR is coded in accordance with International Classification of Diseases (ICD), and hospital contacts were registered using the 8^th^ edition (ICD-8) from 1980 to 1993, and the 10^th^ edition (ICD-10) from 1994 to 2014 (16). In Denmark, all citizens have tax-funded and equal access to public healthcare ensuring a consistent registration of disease in all social classes. This study had an observation period between 1946 and 2016 and a study period between 1980 and 2014. Consequently, patients diagnosed within the study period were included and any registration regarding comorbidities of patients made in the observation period were included.

All patients registered with a HNSCC in DCR in the study period were included, and HNSCC was defined based on ICD-10 codes (**Supplementary Table S1**) and ICD-O-3 morphology codes: 80513, 80523, 80703, 80713, 80723, 80733, 80743, 80753, 80763, 80833, 81203, 81213, 81233. Based on ICD-10 codes, HNSCC were divided into seven topographical groups: Oral cavity, oropharynx, nasopharynx, hypopharynx, sinonasal cavities, larynx, and unspecified sites. The unspecified sites category encompasses overlapping and unspecified sites in the oral cavity and pharynx. Further, additional primary malignancies outside the abovementioned locations were classified in eight topographical groups: Skin, digestive organs, genital organs, respiratory organs, urinary tract, hematolymphoid, breast, and other (**Supplementary Table S2**).

Patient information on vital status, gender, and date of birth in CPR, cause of death in RCG as well as records of hospital contacts in NPR was obtained by individual linkage of their unique civil registration number. Death was considered cancer-specific if one of the registered causes of death was a HNSCC based on ICD-10 codes (**Supplementary Table S1**). The last day of follow-up was December 15^th^, 2016. HNSCC patients were divided into four groups based on treatment modality: Surgery; surgery with adjuvant radiotherapy, chemotherapy, or both; primary radiotherapy, chemotherapy, or both; and unknown. For patients diagnosed between 1980 and 2003, treatment modality was obtained from DCR based on their own classification, and for patients diagnosed between 2004 and 2014, treatment modality was evaluated based on medical and surgical procedures performed in connection with hospital contacts related to the HNSCC diagnosis registered in NPR. The DCR discontinued updating the treatment modality variable after 2003 which is why information on treatment were obtained by two different methods. Each of the included patients was age- and gender-matched with 9–10 control subjects without a HNSCC diagnosis. Control subjects that were censored before the case date of diagnosis was excluded and patients with less than nine matched controls were excluded along with their controls. Data on the control subjects was obtained in the same manner, i.e. from DCR, CPR, and NPR.

CCI was calculated for patients and controls at diagnosis and at 1–10 years after diagnosis. HNSCC diagnosis of the patients was not included in the calculations of CCI. Abbreviations, scoring weights, and corresponding ICD-8 and ICD-10-codes of included comorbid conditions are available in **Supplementary Table S3**. Information on malignancies was obtained from DCR and records of other conditions from NPR. As the NPR contains recorded hospital contacts, the date of an acquired condition is the earliest date that the patient required an inpatient or outpatient hospital visit for the specific condition. Patients and controls were divided into groups with CCI of 0, 1, 2, 3, and ≥4. Further, a comorbidity-age combined risk score was calculated for patients and controls at diagnosis based on CCI and an age score adding an additional point for each decade with 40 years being the zero rank. Patients were divided into four comorbidity-age risk score groups with scores of 2, 3, 4, and ≥5, and into five age groups: <50, 50–59, 60–69, 70–79, and ≥80 years in accordance with the comorbidity-age combined risk score system (9).

### Statistical Analysis

The statistical analysis was performed in R version 3.5.0 (17). The comorbidity burdens of patients and the controls was compared using the R package “fmsb” (18). Odds ratios (OR) for patients to acquire each of the comorbid conditions and a specific additional malignancy compared to controls at date of diagnosis was calculated with the function “fmsb::oddsratio.” Means and 95% confidence intervals of CCI were calculated for subjects at date of diagnosis and at 1–10 years, grouping by gender, age, tumor location, and CCI at date of diagnosis. Unpaired t-tests were carried out to compare means of patients *versus* controls, and males *versus* females. Paired t-tests were carried out to compare means of patients or controls at diagnosis *versus* at 5-year follow-up.

Analysis of overall survival and cancer-specific mortality was carried out using the R packages “Survival” and “Survminer” (19, 20). Univariate and multivariate Cox proportional hazard models were used to compute hazard ratios (HR) for survival including all factors in **Table 1** with the “survival::coxph” function. In the multivariate analysis we adjusted for age, gender, anatomical location, and treatment modality. Kaplan-Meier curves stratified by CCI and comorbidity-age risk score at date of diagnosis was made with the “survival::survfit” and “survminer::ggsurvplot” functions. All patients alive at the last day of follow-up were censored at this date. We considered p-values <0.05 statistically significant.

**Table 1:** Cox regression analysis of survival in head and neck squamous cell carcinoma patients diagnosed between 1980 and 2014.

Characteristics		Overall survival				Cancer-specific mortality			
Group	HNSCC count	Univariate		Multivariate*		Univariate		Multivariate*	
		HR (95% CI)	p	HR (95% CI)	p	HR (95% CI)	p	HR (95% CI)	p
**Total**	25,388 (100%)								
**Gender**									
**Male**	18,403 (72.50%)	Ref	–	Ref	–	Ref	–	Ref	–
**Female**	6,985 (27.50%)	0.94 (0.91–0.97)	0.00	0.85 (0.82–0.88)	0.00	0.93 (0.89–0.98)	<0.001	0.86 (0.82–0.90)	0.00
**Age at diagnosis**									
**<50**	2,908 (11.50%)	Ref	–	Ref	–	Ref	–	Ref	–
**50–59**	6,915 (27.20%)	1.42 (1.35–1.50)	<0.001	1.40 (1.33–1.48)	<0.001	1.34 (1.24–1.44)	<0.001	1.29 (1.19–1.38)	<0.001
**60–69**	8,238 (32.40%)	1.71 (1.62–1.80)	<0.001	1.70 (1.62–1.80)	<0.001	1.35 (1.25–1.45)	<0.001	1.33 (1.24–1.43)	<0.001
**70–79**	5,141 (20.20%)	2.29 (2.17–2.42)	<0.001	2.32 (2.19–2.46)	<0.001	1.59 (1.47–1.72)	<0.001	1.63 (1.50–1.76)	<0.001
**>=80**	2,186 (8.61%)	3.64 (3.41–3.88)	<0.001	3.66 (3.43–3.91)	<0.001	2.59 (2.36–2.83)	<0.001	2.63 (2.39–2.89)	<0.001
**Topography**									
**Larynx**	8,359 (32.90%)	Ref	–	Ref	–	Ref	–	Ref	–
**Oral cavity**	5,876 (23.10%)	1.32 (1.27–1.37)	<0.001	1.67 (1.60–1.74)	<0.001	1.52 (1.44–1.61)	<0.001	1.79 (1.69–1.91)	<0.001
**Oropharynx**	5,803 (22.90%)	1.10 (1.06–1.15)	<0.001	1.35 (1.30–1.41)	<0.001	1.46 (1.38–1.54)	<0.001	1.61 (1.52–1.71)	<0.001
**Hypopharynx**	1,897 (7.47%)	2.22 (2.11–2.35)	<0.001	2.44 (2.31–2.58)	<0.001	3.05 (2.84–3.28)	<0.001	3.14 (2.92–3.38)	<0.001
**Sinonasal cavities**	1,245 (4.90%)	1.06 (0.99–1.14)	0.07	1.14 (1.06–1.21)	<0.001	0.73 (0.65–0.83)	<0.001	0.76 (0.67–0.86)	<0.001
**Nasopharynx**	461 (1.82%)	1.37 (1.23–1.52)	<0.001	1.68 (1.52–1.87)	<0.001	1.18 (0.99–1.40)	0.06	1.35 (1.13–1.60)	<0.001
Unspecified sites**	1,747 (6.88%)	1.42 (1.34–1.51)	<0.001	1.86 (1.75–1.98)	<0.001	1.60 (1.47–1.74)	<0.001	1.91 (1.75–2.08)	<0.001
**Treatment modality**									
**Surgery**	3,365 (13.30%)	Ref	–	Ref	–	Ref	–	Ref	–
**Surgery + RT, CTx or CTx-RT**	6,626 (26.10%)	1.15 (1.09–1.20)	<0.001	1.36 (1.29–1.44)	<0.001	1.40 (1.31–1.51)	<0.001	1.57 (1.46–1.69)	<0.001
**RT, CTx or CTx-RT**	12,578 (49.50%)	1.33 (1.27–1.39)	<0.001	1.70 (1.62–1.79)	<0.001	1.26 (1.18–1.35)	<0.001	1.59 (1.48–1.71)	<0.001
**Unknown**	2,819 (11.10%)	1.85 (1.75–1.96)	<0.001	2.19 (2.07–2.33)	<0.001	2.13 (1.97–2.32)	<0.001	2.49 (2.29–2.71)	<0.001
**CCI at diagnosis**									
**0**	15,743 (62.00%)	Ref	–	Ref	–	Ref	–	Ref	–
**1**	4,393 (17.30%)	1.45 (1.39–1.50)	<0.001	1.34 (1.29–1.39)	<0.001	1.58 (1.49–1.66)	<0.001	1.48 (1.41–1.57)	<0.001
**2**	2,847 (11.20%)	1.55 (1.49–1.63)	<0.001	1.37 (1.31–1.44)	<0.001	1.66 (1.56–1.77)	<0.001	1.53 (1.43–1.63)	<0.001
**3**	1,152 (4.54%)	1.85 (1.73–1.98)	<0.001	1.63 (1.52–1.74)	<0.001	2.21 (2.02–2.41)	<0.001	1.99 (1.82–2.17)	<0.001
**>=4**	1,253 (4.94%)	1.72 (1.61–1.84)	<0.001	1.59 (1.49–1.70)	<0.001	2.23 (2.05–2.43)	<0.001	1.94 (1.78–2.12)	<0.001

HNSCC, Head and neck squamous cell carcinoma; CTx-RT, Radiotherapy, chemotherapy or both; CCI, Charlson Comorbidity Index; HR, Hazard ratio; CI, Confidence interval

*Multivariate Cox regression analysis with covariates gender (male; female), age (<50; 50–60; 60–69; 70-79; ≥80), tumor location (Oral cavity; Oropharynx; Nasopharynx; Hypopharynx; Sinonasal cavities; Larynx; Unspecified sites), and CCI (2; 3; ≥4) at date of diagnosis and at 5-year follow-up.

**Encompasses overlapping and unspecified sites in the oral cavity and pharynx.

## Results

A total of 25,388 HNSCC patients (72.5% male), and 253,368 matched controls were included. The mean age at diagnosis was 63.2 years (95%CI: 63.04–63.33) and the median years of follow-up was 3.0 years (95% CI: 2.93–3.08). Five-year survivors constituted 36.8% (n = 9,350) of all HNSCC patients with a similar gender-distribution (71.2% male). The most prevalent anatomical location was the larynx and oral cavity, accounting for 8,359 (32.9%) and 5,876 (23.1%) patients (**Table 1**).

### Charlson Comorbidity Index at Diagnosis

For patients the mean CCI at diagnosis was 0.81 (95% CI: 0.79–0.82). The comorbidity burden of patients was significantly greater than the comorbidity burden of controls (mean: 0.48; 95% CI: 0.48–0.49) (unpaired t-test of CCI; *p < 0.001*; 95% CI: 0.31–0.34). When including the HNSCC in patient CCI the mean at diagnosis was 2.56 (95% CI: 2.54–2.57) and was also a significantly greater than mean of CCI in controls (unpaired t-test of CCI; *p < 0.001*; 95% CI: 2.06–2.09).

**Figure 1:**
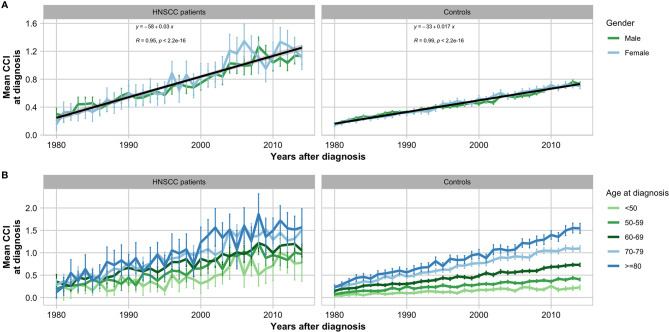
Historical development of Charlson Comorbidity Index (CCI) in head and neck squamous cell carcinoma patients and matched controls in the period 1980–2014 grouped by gender **(A)** and age **(B)** with error bars showing 95% confidence intervals.

During the study period between 1980 and 2014, the mean CCI at diagnosis increased linearly in both patients and controls (**Figure 1**). Fifteen thousand seven hundred forty-three (62.00%) patients had a CCI of 0 at diagnosis, while 4,393 (17.30%) had a CCI of 1; 2,847 (11.20%) had a CCI of 2; 1,152 (4.54%) had a CCI of 3; and 1,253 (4.94%) had a CCI of ≥4 (**Table 1**).

**Figure 2:**
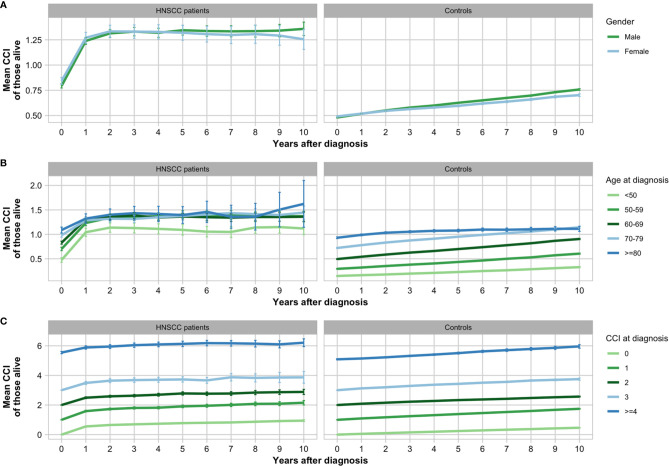
Increase in mean Charlson Comorbidity Index (CCI) of head and neck squamous cell carcinoma (HNSCC) patients and matched controls grouped by gender **(A)**, age **(B)**, and CCI at date of diagnosis **(C)** with error bars showing 95% confidence intervals.

There was a statistical difference between the CCI of male (mean: 0.30; 95% CI: 0.21–0.39) and female (mean: 0.15; 95% CI: 0.07–0.24) patients (unpaired t-test of CCI; p = 0.013; 95% CI: −0.09–−0.10) (**Figure 2**). Further, increased age interval did not correlate directly with increased mean CCI at diagnosis, however, patients aged <50 years (mean: 2.37; 95% CI: 2.39−2.40) had lower CCI at diagnosis than patients in the age groups ≥50 years with means between 2.53 and 2.63. With regards to HNSCC tumor location, the lowest mean CCI at diagnosis was seen in patients with tumors of the sinonasal cavities (mean 2.44; 96% CI: 2.39–2.48) and larynx (mean 2.49; 96% CI: 2.47–2.51). The highest mean CCI was seen in patients with tumors of the hypopharynx (mean: 2.73; 95% CI: 2.67–2.79) and oropharynx (mean: 2.64; 95% CI: 2.61–2.67).

### Comorbid Conditions

The most common comorbid conditions among patients at diagnosis, not including their HNSCC, were additional non-metastatic malignancy (n = 2,772; 10.9%) and cerebrovascular disease (n = 1,962; 7.7%). Similarly, the most common conditions among controls were also non-metastatic malignancy (n = 20,923; 8.3%) and cerebrovascular disease (n = 14,249; 5.6%). Patients were in greater risk of having CCI-related conditions at the date of HNSCC diagnosis compared to controls. Further, the OR of patients having any of the 17 CCI conditions were >1.00 and 13 of these were significant (**Table 2**). Notably, patients had significantly higher odds of having mild liver disease (OR: 6.95; 95% CI: 6.42–7.53; *p < 0.001*) and moderate to severe liver disease (OR: 7.28; 95% CI: 6.14–8.65; *p < 0.001*), as well as non-metastatic malignancy (OR: 1.36; 95% CI: 1.31–1.42; *p < 0.001*) and metastatic malignancy (OR: 4.65; 95% CI: 4.21–5.15; *p < 0.001*).

**Table 2:** Odds ratios of head and neck squamous cell carcinoma patients having each of the Charlson Comorbidity Index conditions compared to the control group.

	All HNSCC patients and matched controls				Five-year survivors and controls alive after 5 years			
	HNSCC count (%)	Control count (%)	OR (95% CI)	*p*	HNSCC count (%)	Control count (%)	OR (95% CI)	*p*
**Comorbid conditions of the Charlson Comorbidity Index**								
Myocardial infarction	1,136 (4.5%)	11,252 (4.4%)	1.01 (0.95–1.07)	0.804	350 (3.7%)	6,690 (3.6%)	1.06 (0.95–1.18)	*0.327*
Congestive heart failure	445 (1.8%)	3,601 (1.4%)	1.24 (1.12–1.37)	*<0.001*	72 (0.8%)	1,348 (0.7%)	1.08 (0.85–1.37)	*0.542*
Peripheral vascular disease	1333 (5.3%)	6,214 (2.5%)	2.2 (2.07–2.34)	*<0.001*	323 (3.5%)	3,099 (1.6%)	2.14 (1.9–2.4)	*<0.001*
Cerebrovascular disease	1962 (7.7%)	14,249 (5.6%)	1.41 (1.34–1.48)	*<0.001*	451 (4.8%)	7,736 (4.1%)	1.18 (1.07–1.3)	*0.001*
Dementia	165 (0.6%)	1,214 (0.5%)	1.36 (1.15–1.6)	*<0.001*	21 (0.2%)	380 (0.2%)	1.11 (0.72–1.73)	*0.631*
Chronic pulmonary disease	1319 (5.2%)	8,901 (3.5%)	1.51 (1.42–1.6)	*<0.001*	308 (3.3%)	4,785 (2.5%)	1.31 (1.16–1.47)	*<0.001*
Connective tissue disease	465 (1.8%)	4,352 (1.7%)	1.07 (0.97–1.18)	0.184	134 (1.4%)	2,689 (1.4%)	1 (0.84–1.2)	*0.962*
Ulcer disease	1517 (6%)	7,911 (3.1%)	1.97 (1.86–2.09)	*<0.001*	378 (4%)	4,828 (2.6%)	1.6 (1.44–1.78)	*<0.001*
Mild liver disease	1047 (4.1%)	1,558 (0.6%)	6.95 (6.42–7.53)	*<0.001*	216 (2.3%)	845 (0.4%)	5.25 (4.51–6.1)	*<0.001*
Diabetes mellitus	677 (2.7%)	6,108 (2.4%)	1.11 (1.02–1.2)	0.012	172 (1.8%)	3,509 (1.9%)	0.99 (0.85–1.15)	*0.873*
Hemiplegia	30 (0.1%)	250 (0.1%)	1.2 (0.82–1.75)	0.35	3 (0%)	133 (0.1%)	0.45 (0.14–1.43)	*0.177*
Chronic kidney disease	273 (1.1%)	1,901 (0.8%)	1.44 (1.27–1.63)	*<0.001*	51 (0.5%)	864 (0.5%)	1.19 (0.9–1.58)	*0.227*
Diabetes mellitus with chronic complications	303 (1.2%)	2,972 (1.2%)	1.02 (0.9–1.15)	0.773	90 (1%)	1,549 (0.8%)	1.17 (0.95–1.45)	*0.144*
Non metastatic malignancy*	2772 (10.9%)	20,923 (8.3%)	1.36 (1.31–1.42)	*<0.001*	700 (7.5%)	11,155 (5.9%)	1.29 (1.19–1.39)	*<0.001*
Moderate to severe liver disease	226 (0.9%)	312 (0.1%)	7.28 (6.14–8.65)	*<0.001*	32 (0.3%)	133 (0.1%)	4.86 (3.3–7.16)	*<0.001*
Metastatic malignancy	582 (2.3%)	1,273 (0.5%)	4.65 (4.21–5.13)	*<0.001*	174 (1.9%)	544 (0.3%)	6.55 (5.51–7.78)	*<0.001*
AIDS	31 (0.1%)	119 (0%)	2.6 (1.75–3.86)	*<0.001*	7 (0.1%)	75 (0%)	1.88 (0.87–4.08)	*0.11*
Additional tumor (Mal*) categorized by anatomical location**								
Skin	995 (3.9%)	8,225 (3.2%)	1.22 (1.14–1.3)	*<0.001*	286 (3.1%)	4,812 (2.6%)	1.2 (1.07–1.36)	*0.003*
Respiratory organs	224 (0.9%)	785 (0.3%)	2.86 (2.47–3.32)	*<0.001*	21 (0.2%)	173 (0.1%)	2.45 (1.56–3.85)	*<0.001*
Digestive organs	353 (1.4%)	2,786 (1.1%)	1.27 (1.13–1.42)	*<0.001*	65 (0.7%)	1,285 (0.7%)	1.02 (0.79–1.31)	*0.88*
Genital organs	372 (1.5%)	3,740 (1.5%)	0.99 (0.89–1.11)	*0.891*	102 (1.1%)	1,781 (0.9%)	1.16 (0.95–1.41)	*0.157*
Breast	243 (1%)	2,130 (0.8%)	1.14 (1–1.3)	*0.054*	56 (0.6%)	1,216 (0.6%)	0.93 (0.71–1.21)	*0.583*
Urinary tract	133 (0.5%)	1,021 (0.4%)	1.3 (1.09–1.56)	*0.004*	27 (0.3%)	482 (0.3%)	1.13 (0.77–1.66)	*0.54*
Hematolymphoid	71 (0.3%)	732 (0.3%)	0.97 (0.76–1.24)	*0.793*	12 (0.1%)	281 (0.1%)	0.86 (0.48–1.53)	*0.61*
Other	47 (0.2%)	366 (0.1%)	1.28 (0.95–1.74)	*0.109*	13 (0.1%)	182 (0.1%)	1.44 (0.82–2.53)	*0.204*

HNSCC, Head and neck squamous cell carcinoma; OR, Odds ratio; CI, Confidence interval. *Non-metastatic malignancy does not include tumors of the oral cavity, pharynx, sinonasal cavities, and larynx (ICD-10: C01–C06, C09–C14, C30–C32). **ICD-10 codes used for categorizing anatomical location of additional tumors outside the oral cavity, oropharynx, nasopharynx, hypopharynx, sinonasal cavities, and larynx can be seen in.

**Figure 3:**
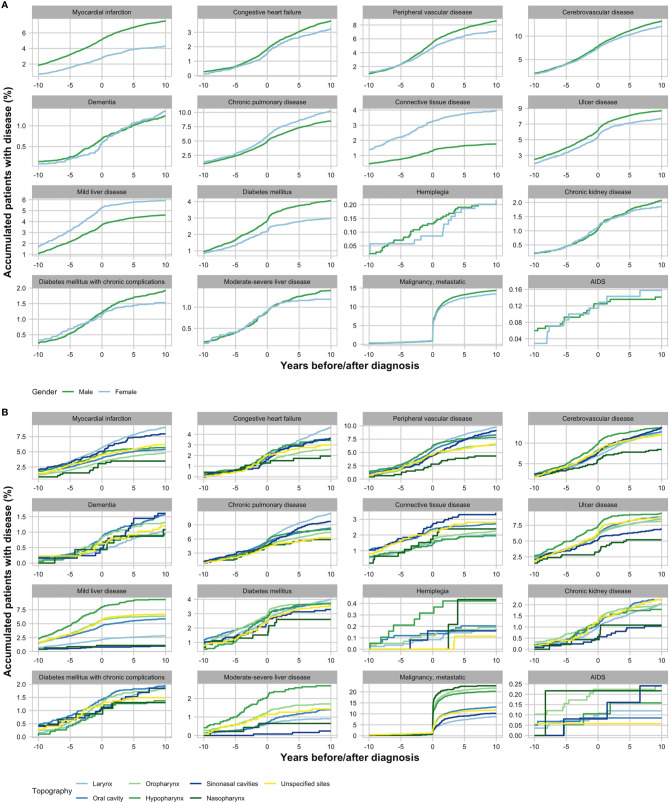
Acquisition of Charlson Comorbidity Index (CCI) conditions in head and neck squamous cell carcinoma patients from 10 years before until 10 years after diagnosis grouped by gender **(A)** and anatomical location of tumor **(B)**.

The most common additional malignancies among patients were cancers of the skin (n = 995; 3.9%), genital organs (n = 353; 1.4%), and digestive organs (n = 353; 1.4%) (**Table 2**). In the control group, the most common malignancies were also cancers of the skin (n = 8,225; 3.2%), genital organs (n = 3,740; 5.4%), and digestive organs (n = 2,786; 1.1%). Compared with controls, patients were at higher risk of malignancies in the respiratory organs (OR: 2.86; 95% CI: 2.47–3.32; *p < 0.001*) and digestive organs (OR: 1.27; 95% CI: 1.13–1.42; *p < 0.001*). Esophageal neoplasms (ICD-10: C15) accounted for 18.7% (n = 66) of cases’ digestive organ tumors and 2.3% (n = 65) controls’ digestive organ tumors. Myocardial infarction and diabetes were more common among male patients than female patients, whereas mild liver disease and connective tissue disease were more common among females (**Figure 3A**).

### Acquisition of Comorbidities After Diagnosis

In the years following the HNSCC diagnoses, CCI and thus the prevalence of comorbid conditions increased among both patients and matched controls (**Figures 2** and **3**). The increase in CCI between the date of HNSCC diagnoses and 5 years after was significant in both patients (paired t-test; *p < 0.001*; 95% CI: 0.75–0.82) and controls (paired t-test; *p < 0.001*; 95% CI: 0.27–0.27). In patients, the highest increase of CCI was seen in the first year after diagnosis with a mean of 0.44 (95% CI: 1.22–1.28) and the percentage of patients with metastatic malignancy increased drastically immediately following the diagnosis. This was especially the case in pharyngeal HNSCC (**Figure 3B**). Preceding the first 2 years after diagnosis the curve flattened and the mean CCI only changed between means of −0.01–0.01. In controls, contrarily, CCI increased linearly over the 10 years by an annual mean of 0.03 (95% CI: 0.02–0.03).

At 5-year follow-up, the CCI among 5-year survivors (mean: 1.34; 95% CI: 1.3–1.38) and alive controls (mean: 0.86; 95% CI: 0.85–0.86) remained significantly different (unpaired t-test; *p < 0.001*; 95% CI: 0.68–0.76). Generally, a lower prevalence of comorbid conditions at diagnosis was seen in 5-year survivors compared to all patients with the most common conditions remaining the same (**Table 2**).

### Comorbidity-Related Survival

In the multivariate analysis adjusting for age, gender, treatment modality, and anatomical location, the increase in CCI was significantly associated to decrease in survival probability at date of diagnosis and 5 years later (**Table 1**). In the multivariate Cox regression analysis of overall survival, patients with CCI of 1, 2, 3, and ≥4 at date of diagnosis had HR of 1.34 (1.29–1.39; p < 0.001), 1.37 (1.31–1.44; p < 0.001), 1.63 (1.52–1.74; p < 0.001), and 1.59 (1.49–1.70; p < 0.001) compared to patients with CCI of 0.

In the multivariate Cox regression analysis of cancer-specific mortality, patients CCI of 1, 2, 3, and ≥4 at date of diagnosis had HR of 1.48 (1.41–1.57; p < 0.001), 1.53 (1.43–1.63; p < 0.001), 1.99 (1.82–2.17; p < 0.001), and 1.94 (1.78–2.12; p < 0.001) compared to patients with CCI of 0.

**Figure 4:**
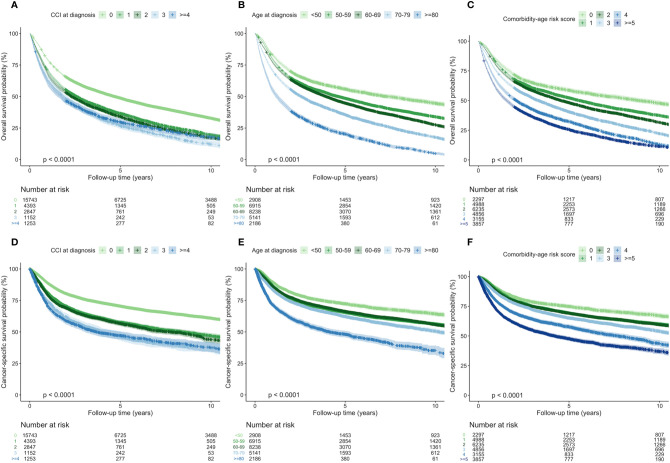
Survival curves with 95% confidence intervals diagnosis patients with head and neck squamous cell carcinoma in all patients stratified by Charlson Comorbidity Index (CCI) at diagnosis, age at diagnosis, and comorbidity-age risk score showing overall survival **(A–C)** and cancer-specific mortality **(D–F)**.

In Kaplan-Meier curves, decreased overall survival probability was significantly associated with high CCI at date of diagnosis (**Figure 4A**). However, a stronger correlation was seen when stratifying by age (**Figure 4B**) and comorbidity-age risk score (**Figure 4C**). The same trends were seen regarding the cancer-specific mortality (**Figures 4D–F**).

## Discussion

This nationwide study reports that HNSCC patients in Denmark diagnosed between 1980 and 2014 was more at risk of having the CCI-related conditions compared to a sex- and age-matched control group with OR of all CCI-related conditions being >1.00.

Historically, the overall mean CCI at date of diagnosis increased during the study period 1980–2014 in both patients and controls (**Figure 1**). This tendency could be explained by prolonged lifespan and the global increase of morbidity observed in conditions including cancer, liver disease, diabetes mellitus, cardiovascular disease, kidney disease, and dementia (21, 22). Another likely contributing factor is the increase in registrations during the observation period as the NPR has been continuously extended since 1977 (16). Further, since this study investigates comorbidities over a long period, it is very likely that the risks associated with the morbidities has changed due to alterations in diagnostics and treatment procedures. For instance, the mortality of ulcer disease and cardiovascular disease had decreased in Denmark during the study period (23, 24). This could be the focus of an interesting future project.

The CCI at diagnosis proved to be an independent prognostic factor in HNSCC patients (**Table 1**). This was even stronger when looking solely at cancer-specific mortality. These findings correlate with earlier findings that CCI is highly associated with mortality in the general population in Australia and has proved to be a strong prognostic factor for survival in HNSCC patients (10, 25). In patients, the highest increase of CCI was seen in the first year after diagnosis, however, in controls CCI increased steadily over the 10 years (**Figure 2**). An obvious reason for this is the high increase of diagnoses of metastatic malignancy after diagnosis (**Figure 3**). Another reason could be comorbidities related to treatment modality, e.g. postoperative complications. As HNSCC is associated with lifestyle behavior, it is feasible that the rapid increase of CCI within the first year may also be a result of accumulated undiagnosed comorbidities that were then discovered in connection with or immediately following the HNSCC diagnosis due to increased contact with the Danish health care system. A reason for the reluctant changes in patients’ CCI beyond the first 2 years could be that the mean CCI is evaluated in patients alive, and since 63.2% of patients died within the first 5 years, these patients do not contribute to the mean CCI at 5-year follow-up. A plausible contributing factor may also be the limited observation period for patients diagnosed late in the study period. Finally, it could be explained by the fact that new diagnoses within the same CCI-related condition category as listed in **Supplementary Table S3** does not increase the calculated CCI.

Of the CCI-related conditions, liver disease, and cancers of liver and lungs are especially associated with smoking and alcohol (26–29). Because HNSCC is related to lifestyle behavior that give rise to disease of multiple organs, this might explain the higher odds of patients having mild to severe liver disease as well as non-metastatic and metastatic malignancy that was observed in this study (**Table 2**). Previous findings have shown tumors of lung and esophagus to be the most common secondary primary tumor in HNSCC patients apart from tumors in the head and neck region (30). In correlation with this, we found that patients had almost three times higher odds of having a cancer of the respiratory and intrathoracic organs at diagnosis. Though we did observe that patients had higher odds of having a digestive tumor, the difference between patients and controls were not as big as for the respiratory tumors. This may be explained by the fact that tumors of the digestive organs cover many anatomical locations. In coherence, we observed that tumors of the digestive organs in patients more often originated from the esophagus compared to controls. A contributing factor could be that patients died before the tumor of the digestive system was acquired and/or diagnosed. The exceptionally rapid increase of metastatic malignancy in patients seen in **Figure 3** can be explained in part by their HNSCC, and in part by their increased likelihood of acquiring an additional malignancy. Thus, the increase in diagnosed metastatic malignancies after primary diagnosis could be directly caused by the later discovery of metastases originating from the HNSCC or an additional acquired tumor. In addition to mean CCI at diagnosis being highest in patients with hypopharynx and oropharynx tumors, patients with pharyngeal tumors had the largest increase in metastatic malignancy diagnoses compared to the other locations. This disunity between the pharyngeal HNSCC and the rest continued 10 years after diagnosis and could explain the high HR in patients with tumors of the hypopharynx and nasopharynx, compared to patients with larynx cancer (**Table 1**).

Females among 5-year survivors had lower CCI at diagnosis compared to males, which may be attributable to the fact that females are less likely to demonstrate risk-associated behavior such as tobacco use and alcohol consumption (31–33). These behavioral differences may also, together with the high CCI at diagnosis, explain the higher mortality observed in males compared to females (**Table 1**).

Acquisition of CCI-related conditions did not seem to correlate with age, however, we observed that both CCI and age influenced survival (**Table 1**). Increasing age significantly correlated with increasing HR in both the univariate and multivariate analyses, and the resulting HR indicates that age at diagnosis contributed more to survival than comorbidity at diagnosis. This is controversial to previous findings showing that comorbidities and frailty have more influence than age in determining 3-month postoperative mortality in geriatric cancer patients (34). In head and neck cancer patients, frailty strongly contributes to increased mortality and, in coherence with our findings, increases with age (35). Since we observed that age and comorbidity-age risk scores were stronger predicters of survival than CCI in the Kaplan Meier curves (**Figure 4**), our findings suggest that age is a vital part of the predictive value of the comorbidity-age risk score and thus CCI should be combined with age.

The fact that this registry-based study includes the entire Danish population is a clear strength as it avoids the selection bias that most retrospective studies are prone to. Further, this study provides a picture of the comorbidity burden of patients across social classes as the tax-funded Danish health care system facilitates registration of disease independent of social class. However, this report is subject to other common biases associated with registry-based studies such as discrepancies between clinics and clinicians, as well as changes in clinical practice over time, e.g. regarding the use of diagnosis codes (i.e. ICD-8 and ICD-10). In general, the reporting of comorbidities among patients and controls was limited by the last day of follow-up in 2016. Another limitation is that the registration practice to the NPR as well as the general risk of diseases and the associated mortality has likely changed during the 35-year study period.

The use of DCR to report malignancy had the advantage that DCR includes morphology and takes misdiagnosing into consideration, however, had the disadvantage that registration was not mandatory before 1987 (13). Although registry-based studies have limitations, we expect our use of NPR to report the non-malignant CCI-related conditions to be precise, as the use of CCI in NPR has previously been shown to have an overall predictive value of 98% (11). As seen in previous comorbidities studies (36), the administrative data used in this study does not provide information on the exact dates that the comorbid conditions first appear. Rather, this study gives an insight into the prevalence of comorbidities that were serious enough to require at least one inpatient or outpatient hospital visit. This means that the comorbidity burden may be even stronger than shown in this study.

The focus on HNSCC in this study provides a clear picture of the patient group. Due to the fact that patients with cancers of the salivary and thyroid glands are associated with gender disparity and radiation rather than tobacco use and alcohol consumption it was a strength to not include patients with these cancers despite the fact that they are affiliated with head and neck surgery in Denmark (37–39).

The fact CCI did not include the HNSCC diagnoses of patients was ideal when comparing patient groups, as additional non-metastatic malignancies in patients before or after the HNSCC diagnosis could weigh in the CCI score.

In conclusion, we found that CCI is an independent prognostic factor in HNSCC patients, and the highest increase of CCI was seen in the first year after diagnosis. Further research should investigate the impact of lifestyle behavior, hospitalization duration, number of hospital contacts before and after diagnosis, and treatment modality on CCI-related survival and examine the nature of additional tumors among HNSCC patients.

## Data Availability Statement

Data used in this study is available upon request to the corresponding author, however, requires specific permission from the Danish Patient Safety Authority and the Danish Data Protection Agency which can be applied for. Requests to access these datasets should be directed to christian.groenhoej@regionh.dk..

## Ethics Statement

Permission to analyze the retrospective data included in this study was provided by Danish Patient Safety Authority and the Danish Data Protection Agency. The study was performed in accordance with the Declaration of Helsinki.

## Author Contributions

CG, KJ, JJ, and EK devised the project and the main conceptual ideas. EK carried out the computational and statistical analysis. IW and CV advised the project with regards to head and neck surgery. GL advised the project with regards to pathology. EK, CG, and JJ wrote the manuscript. All authors provided critical feedback and helped shape the analysis. CV and CG supervised the project. All authors contributed to the article and approved the submitted version.

## Conflict of Interest

The authors declare that the research was conducted in the absence of any commercial or financial relationships that could be construed as a potential conflict of interest.
