# Supplementary material for: The Impact of Comorbidity on Survival in Patients With Head and Neck Squamous Cell Carcinoma: A Nationwide Case-Control Study Spanning 35 Years
Source: Front Oncol. 2021 Feb 17;10:617184. doi: 10.3389/fonc.2020.617184 (PMC7928275; doi:10.3389/fonc.2020.617184)
Supplement: Supplementary file 1 [file DataSheet_1.pdf]

Supplementary Table S1 *ICD-10 codes and corresponding anatomical location of head and neck squamous cell carcinoma*

Anatomical location	ICD-10 codes
Oral cavity	C02.1-C02.3; C03-C04; C05.0; C06
Oropharynx	C01; C02.4; C05.1-C05.2; C09-C10
Nasopharynx	C11
Hypopharynx	C12-C13
Sinonasal cavities	C30-C31
Larynx	C32
Unspecified sites*	C02.8-C02.9; C05.8-C05.9; C14

* Encompasses overlapping and unspecified sites in the oral cavity and pharynx.

Supplementary Table S2 *ICD-10 codes and corresponding anatomical location of malignancies that are not regions of head and neck squamous cell carcinoma tumors*

Anatomical location	ICD-10 codes
Skin*	C00; C43-C44
Respiratory organs**	C33-C39
Digestive organs	C15-C26
Genital organs	C51-C58; C60-C63
Breast	C50
Urinary tract	C64-C68
Hematolymphoid	C81-C96
Other***	C07-C08; C40-C41; C45-C49; C69-C75

* Includes neoplasms of lip (ICD-10: C00)

** Includes neoplasms of lower respiratory and intrathoracic organs, excludes neoplasms of upper respiratory organs, i.e. head and neck cancer with the anatomical location of sinonasal cavities and larynx (ICD-10: C30-C32).

*** Includes neoplasms of salivary gland and thyroid glands (ICD-10: C07-C08; C73).

Supplementary Table S3 *Charlson Comorbidity Index conditions, ICD codes and weights*

Condition	Abbr.	W	Reg.	ICD-8 codes	ICD-10 codes
Myocardial infarction	MI	1	NPR	410	I21; I22; I23
Congestive heart failure	CHF	1	NPR	427.09; 427.10; 427.11; 427.19; 428.99; 782.49	I50; I11.0; I13.0; I13.2
Peripheral vascular disease	PVD	1	NPR	440-445	I70; I71; I72; I73; I74; I77
Cerebrovascular disease	CVD	1	NPR	430-438	I60-I69; G45; G46
Dementia	Dem	1	NPR	290.09-290.19; 293.09	F00-F03; F05.1; G30
Chronic pulmonary disease	CPD	1	NPR	490-493; 515-518	J40-J47; J60-J67; J68.4; J70.1; J70.3; J84.1; J92.0; J96.1; J98.2; J98.3
Connective tissue disease	CTD	1	NPR	135.99; 446; 712; 716; 734	M05-M06; M08-M09; M30-M36; D86
Ulcer disease	UD	1	NPR	530.91; 530.98; 531-534	K22.1; K25-K28
Mild liver disease	MLD	1	NPR	571; 573.01; 573.04	B18; K70.0-K70.3; K70.9; K71; K73; K74; K76.0
Diabetes mellitus	DM	1	NPR	249.00; 249.06; 249.07; 249.09; 250.00; 250.06; 250.07; 250.09	E10.0; E10.1; E10.9; E11.0; E11.1; E11.9
Hemiplegia	Hem	2	NPR	344	G81; G82
Chronic kidney disease	CKD	2	NPR	403; 404; 580-583; 584; 590.09; 593.19; 753.10-753.19; 792	I12; I13; N00-N05; N07; N11; N14; N17-N19; Q61
Diabetes mellitus with chronic complications	DMC	2	NPR	249.01-249.05; 249.08; 250.01-250.05; 250.08	E10.2-E10.8; E11.2-E11.8
Malignancy, non-metastatic*	Mal	2	DCR	140; 142; 150-159; 162-194; 200-207; 275.59	C00; C07-C08; C15-C29; C33-C75; C81-C96
Moderate to severe liver disease	SLD	3	NPR	070.00; 070.02; 070.04; 070.06; 070.08; 456.00-456.09; 573.00	B15.0; B16.0; B16.2; B19.0; K70.4; K72; K76.6; I85
Malignancy, metastatic	MalM	6	DCR	ICD-8 codes not used in DCR.	C76-C80
AIDS	AIDS	6	NPR	079.83	B21-B24

Reg: Register used; NPR: The Danish National Patient Register Da DCR: The Danish Cancer Register; Abbr: Abbreviation; W: Charlson Comorbidity Index (CCI) weight

* Encompasses the following comorbid conditions: 1) Solid tumor; 2) Leukemia; and 3) Lymphoma
